# Odontological Society of Pennsylvania

**Published:** 1892-03

**Authors:** 


### ODONTOLOGICAL SOCIETY OF PENNSYLVANIA.

At the regular monthly meeting, held January 11, 1892, the President, Dr. Jack, introduced the essayist of the evening, Dr. G. W. Weld, of New York City, who read a paper entitled "The Syrup of Iron Chloride."

(For Dr. Weld's paper, see page 161.)

This was followed by a discussion, which was opened by Professor A. H. Elliott, of New York, who said: I first became interested in this subject from some experiments of my friend, Dr. Weld, five or six years ago, when he brought to my notice the astonishing fact that he could neutralize the acid of the salt of iron without precipitating it. At first I was inclined to laugh at him. After he had demonstrated to me that it was so I went to his office and saw some experiments he was making with Vichy water, and he was adding this in very liberal quantities to tincture of muriate of iron without precipitating it. It certainly was a fact, but it was rather contrary to my preconceived notions of chemical reactions. Nevertheless he interested me in the action of tincture of iron and acids upon the teeth, and I made an analysis of the enamel and arrived at its chemical structure, and I also undertook experiments with regard to the action of acids on the teeth, and this led me to think of the possible union that could take place between Vichy water and the iron salt which he had certainly neutralized.

All the authorities upon the preparation of the tincture of chloride of iron are agreed that you could not have a tincture of chloride of iron minus the free acid without precipitating it.

It has been concluded that free acid in conjunction with alcohol formed ethyl chloride which forms in tincture of chloride of iron and thus eliminates the acid, but that is evidently not so. Dr. Weld's experiments with the teeth and also in connection with carbonate of lime and other substances have proved beyond a question that acid is still present in tincture muriate of iron.

In the action of acid upon the teeth, certain modifying circumstances can take place. Suppose we get rid of all the hydrochloric acid, and that we have nothing but the pure perchloride of iron left with the minimum amount of acid. If you place any substance in it that is capable of reacting with it, as carbonate of lime, which is part of the enamel of the teeth, you could readily get chloride of calcium from the carbonate of lime and you would get basic carbonate of iron. So if we could get tincture muriate of iron without acid that would not precipitate, we should be all right; that is, get it free from acid and not precipitate basic salts of iron; we must get the fluid into the condition that it will not allow the salts of iron to diffuse. That is, it should make them adhere to the teeth and thus protect them, or if this will not do we must introduce something else to prevent it. The doctor has not only succeeded in eliminating the acid, but he has succeeded in eliminating the action of the chloride of iron itself, or rather prevented the action of chloride of iron upon the enamel of the teeth by having there a dense syrup in which the iron compounds are partially soluble.

While he was speaking I made one or two notes: "The reason that the tooth is not acted upon by the concentrated tincture of muriate of iron, he showed, was due to the fact that alcohol present in it prevents diffusion of the iron salts into the fluid." With regard to acid saliva, I am not a physiological chemist, but I think of these things in connection with chemical facts, and it appears to me that in every case where the saliva is said to have been acid, it is under circumstances where no precaution has been taken to get rid of the food adhering to the mouth, and it is not improbable that after it has passed the mouth, the residue has become the victim of bacteria. Fermentation has been set up by the substances that have been left in the food adhering to the mouth, and that is probably the cause in many cases of the apparently acid reaction of the saliva.

There was a case cited where a number of cows had been fed on distillery slops. That is acid. We have had a great many cases of that kind in New York. The slops were often acid, and of course, if a cow has been living on that food, why should not the interior of the mouth be of the same character, unless it be thoroughly washed out?

*Dr. Dwinelle*, New York.—Probably this question influences or affects no profession more than it does ours. It is well known, or used to be with me, that the mere tincture of iron was one of the most important tonics we had to deal with, and in a short time it has been eliminated from that as the great objection,—its action upon the teeth,—and we are trying to resort to mechanical and other means, such as tubes, quills, etc., to avoid the hydrochloric acid coming in contact with the teeth. So much has been this objection that in many cases they do not use it at all; it has been almost abandoned by the profession, but since this preparation has come to the front we are encouraged to believe we can resort to it and secure the advantages we have derived from it heretofore. I trust that will be the case.

Some singular chemical facts have come to our knowledge through Dr. Weld's paper,—almost an anomaly, almost a contradiction; in fact, it is somewhat like the homœopathic theory,—the weaker a thing is the stronger it is.

We have seen by experiments that when the teeth have been in conjunction with pure hydrochloric acid the action is slow, but reduce it by eight times its volume of water and it at once becomes potent, powerful, and effectual. Dr. Weld must be the first one who made that discovery. The elimination of the free acid, or causes of acid, removes one of the objections so far as the teeth are concerned; furthermore, the removal of the acid renders it more acceptable to the stomach and the mucus membranes at large; in eliminating it in this way it is more readily tolerated, and becomes an agreeable medicine to take.

This subject that Dr. Weld has given us is a text we can preach upon at our leisure, and can make very profitable for thought. There is no profession that it interests more than our own. It is a most interesting subject, and we will all profit by it.

*Dr. Mills*, New York.—The paper has interested me very much and I think I cannot do better than to bring to the notice of the meeting something that has attracted my attention in several journals; among the rest was the *Commercial Advertiser*, and also an article in the *North American Review*, in which is given the past and future of medicine, and the writer arraigns medicine for the slow progress made. Among the other criticisms are some things I think might be profitably brought to our attention in the dental profession. He says that among the things that have stood in the way of progress and scientific action among medical men is the want of mental culture, and a lack of scientific ambition to investigate special subjects. He speaks of the few remedies that have any peculiar bearing upon the practice in special diseases. It seems to me that this paper has impressed itself on my mind in this direction. Dr. Weld has exhibited here his ability to prove that he has some scientific ambition. We ought to congratulate ourselves that we have one among us who can bring such a paper as this to our attention. He has brought forth a specific remedy, and by taking away the hydrochloric acid he is able to take away the deleterious effect of the action upon the enamel.

*Dr. Jas. Truman.*—I was extremely interested in the doctor's former paper read before the Odontological Society of New York, and was especially gratified that he had taken up this subject and had carried it forward to the extent he had at that time. He has shown us to-night the possibility of using chloride of iron without injury. It is a subject that has claimed more interest in the dental profession than it has, unfortunately, in the medical. Every individual in practice has known of the disastrous results that have followed the use of chloride of iron, many cases occurring where our best efforts have been neutralized by its use. In my own experience I might recite many very serious cases where whole sets of teeth have had portions of the anterior surfaces destroyed by a treatment of chloride of iron. It has become, therefore, a matter of vital importance to us as dentists to try to eliminate this agent from the medical profession, or, to do as Dr. Weld has, give them something they can use without injury.

There is really nothing in this paper to antagonize, and therefore it is not readily discussed without taking up the entire subject; but there is one point I would like Dr. Weld in his response to reply to, and that is, that if this agent or preparation that he has made passes into the stomach and there undergoes a change back to hydrochloric acid, as I understand him to say, whether there was not a danger of possible regurgitation and secondary acid action on the teeth?

I was struck with the remark of Professor Elliot, that the saliva of animals will become acid by the use of certain still-slops. I remember a number of years ago, in the western part of New York State, where I was then residing, that I was brought in contact more or less with the animals that were kept at the distillery. Large droves of hogs were fed on the still-slops to the destruction of their teeth, and in a very few weeks these were entirely eroded, and that to such an extent as to be almost useless. I have noticed the same effect from still-slops in other directions. I do not believe, with Dr. Elliot, that the saliva of animals is ever acid, or at least if it is acid it must be on exceptional occasions. I go further than that, and am prepared to assert that human saliva is very rarely acid. I have not found it so in my tests of the secretions of the mouth, nor is it alkaline, but gives a neutral response. It was on that basis I was led to entertain the hypothesis that the oral secretions during the active period of the twenty-four hours, or during the day-time, could not possibly act upon the teeth. I therefore determined to extend the testing to the period of absolute rest, or during the night, and it was my experience in repeated observations that the saliva during these hours gave an acid reaction.

Now, I agree fully with Professor Elliot in regard to the possibility of foods fermenting by being left in the mouth. If any one will test the mouth in those quiet places, such as cavities that are outside the active rush of the salivary fluids, they will find they get an acid reaction. I hope this preparation of Dr. Weld's will be adopted, but there seems to be in the medical profession a general antagonism to the opinion of dentists in regard to these matters.

*Dr. Faught.*—The subject is one in which I have been long interested from a personal relationship. While I wish to state the result of my observations here to-night, I do not cite these cases as being antagonistic at all, but it has been to me a matter of deep interest. I have had a great deal to do with the use of the ordinary tincture of iron in the case of my children. I do not find in their mouths, from its use through a period of years, any more, nor indeed as much, dental caries occurring as it has been my custom to observe in the mouths of other children. I state this because we have not taken precautions, for reasons of my own, as to the use of tubes or quills.

I have been in the habit for some time of taking medical care of my children to a certain degree. For a long period the older child has been subject to recurrent attacks of tonsillitis, which would occur through the winter-time. I painted the tonsils with tincture of iron in dilution of about half water, using it quite frequently, as often as the urgency of the case required. I have used it at all hours of the day and night, and have made free use of the iron internally, giving it in large doses up to the limit, and so with the youngest child for other conditions, but in no instance have I found the condition of the mouths show any evidence of undue breaking down or disintegration of the teeth. And I have not taken preventive measures. There may be reasons which overcome acid reactions, and perhaps the tincture of iron is very carefully prepared; I will say that it is not the tincture of iron ordinarily obtained from drug-stores, and it may not be quite so acid. I merely state these facts because it has been to me a matter of considerable interest and wonder, extending, as it has, through a period of years.

Dr. Weld, in answering Dr. Faught, remarked: Of course, when the modified tincture, so to speak, passes through the mouth into the stomach, it combines with more acid and is likely restored to its former condition. The hydrochloric acid which it meets in the stomach is very small in quanity, and it could not be very much changed. My idea in speaking of that was merely to illustrate that after it passed the mouth and into the stomach its acidity would be increased rather than decreased, and that on account of that increase it would, perhaps, be more likely to be diffused through the system than otherwise. In other words, if we assume it was taken in the stomach, and instead of the acid increasing it should decrease, we would probably get precipitation. We would have an emasculated preparation of iron which would not be diffused. We know there are various preparations of iron which have been put upon the market, and a number of other preparations which are not easily changed when they reach the stomach.

One thing in connection with the chloride of iron is, that when it enters the stomach it is more diffusible than other preparations. As stated in my paper, physicians and practitioners have observed that it had a quicker and better effect than most all other forms of iron. Unfortunately, as you all know, we can get very few children, with, perhaps, the exception of my friend's children, to take that form of iron. It makes but little difference whether it is the children of the wealthy or of the slums or dispensaries. The poor woman says to the physician, "My child will not take that iron," and the wealthy woman's child objects in the same way, and if they take it, it is with a great degree of reluctance. Dr. Winters, a friend of mine in New York, is trying it in cases of diphtheria. He states that after it has been used a certain length of time it excites nausea and vomiting.

I wish to emphasize here a matter which I mentioned before the Odontological Society of New York; that is, that I believe the margin of the gums, as a rule, retains acid longer than any of the other surfaces of the mucous membranes of the mouth, forming a sort of sack that holds it, and that not infrequently if a piece of delicate litmus paper is put to the margin, you get the color indicating an acid present. If the mouth be thoroughly rinsed, there is no acid. If a little acid be taken in the mouth again, and the mouth be rinsed and the litmus paper applied, you get no acid; but at the neck of the tooth you have that reaction. I am not prepared to say at present whether the retention of acid at the margin of the gums can affect the teeth in any way. I have not studied it, but I think there is something in it worth Dr. Kirk's consideration. I am very sorry that that gentleman is not here, and especially so to know that sickness prevents him.

I do not know that I can add anything to what has been said. There are people who go through the world with iron constitutions, and are not affected by alcohol or anything else; and there are some children whose teeth are exempt from acid, and I am glad to know that that is the case with my friend's children. There are chemical laws governing the action of acids on carbonate of lime wherever it is, and the law is unchangeable. It will attack it at once, and all the washing you can do afterwards does not remove the first contact.

*Professor Elliot.*—I once made experiments with some enamel prepared for me from a large number of teeth,—twenty-nine, I think, the doctor said,—from different patients, and I found that a mere immersion in a weak six-per-cent. solution of acetic acid—simply dipping the enamel in and removing it—reduced the weight of the teeth eight per cent.

*Dr. Dwinelle.*—I believe the fact of the deleterious effect of hydrochloric iron upon the teeth has been known and recognized almost as long as medicine has been practised. I believe there is no doubt about it, although there may be exceptions to the rule; but the fact itself remains the same and impregnable.

Dr. Truman said he could not help admiring the courage of Dr. Faught in giving his children chloride of iron in the way he did, though the courage was not to be commended. If a microscope be used, it will be seen how little acid, it takes to dissect out the enamel,—one part in ten of water. The action of hydrochloric acid is readily seen in sections, cutting it away. It is the practice we have always adopted in dissecting out this tissue. Now, if so small an amount will do it, how could Dr. Faught use chloride of iron on his children's teeth *ad libitum*, without producing more or less injury to the teeth?

Dr. Faught explained that he had merely mentioned the case because it was of especial interest to him, and stated that any one who cared to see the children might do so.

Dr. Weld asked if it had been applied locally or systemically, to which Dr. Faught answered that it had been used in both ways. Dr. Faught stated, in answer to Dr. Truman's surprise that an intelligent dentist should use acid so freely, that he had the personal and careful and constant inspection of the teeth, and should have desisted at once upon the appearance of injurious effects.

Professor Elliot asked if he knew the origin of the tincture of iron used, stating that in the College of Pharmacy in New York he would frequently have people come to him and ask if preparations they submitted were up to the standard, and that if Dr. Faught would examine the tincture of iron he had used he would be surprised at the strength. Tincture of iron should contain $7\frac{8}{10}$ per cent. perchloride of iron. Some tincture of iron is very mild as compared with others. Some almost fume from the excessive quantity of acid. Our friend may have gotten hold of some of the mild variety, and it might have been made by some conscientious druggist who prepared his own preparations, and who took care to reduce the iron.

Dr. Faught stated in reply that the iron he had used was procured in every instance from a personal friend,—one of the heads of a department in John Wyeth & Co.'s,—who prepared it for him expressly.

